# Spatial and temporal EEG dynamics of dual-task driving performance

**DOI:** 10.1186/1743-0003-8-11

**Published:** 2011-02-18

**Authors:** Chin-Teng Lin, Shi-An Chen, Tien-Ting Chiu, Hong-Zhang Lin, Li-Wei Ko

**Affiliations:** 1 Brain Research Center, National Chiao-Tung University, Hsinchu, Taiwan; 2 Department of Electrical Engineering, National Chiao-Tung University, Hsinchu, Taiwan; 3 Department of Biological Science and Technology, National Chiao-Tung University, Hsinchu, Taiwan

## Abstract

**Background:**

Driver distraction is a significant cause of traffic accidents. The aim of this study is to investigate Electroencephalography (EEG) dynamics in relation to distraction during driving. To study human cognition under a specific driving task, simulated real driving using virtual reality (VR)-based simulation and designed dual-task events are built, which include unexpected car deviations and mathematics questions.

**Methods:**

We designed five cases with different stimulus onset asynchrony (SOA) to investigate the distraction effects between the deviations and equations. The EEG channel signals are first converted into separated brain sources by independent component analysis (ICA). Then, event-related spectral perturbation (ERSP) changes of the EEG power spectrum are used to evaluate brain dynamics in time-frequency domains.

**Results:**

Power increases in the theta and beta bands are observed in relation with distraction effects in the frontal cortex. In the motor area, alpha and beta power suppressions are also observed. All of the above results are consistently observed across 15 subjects. Additionally, further analysis demonstrates that response time and multiple cortical EEG power both changed significantly with different SOA.

**Conclusions:**

This study suggests that theta power increases in the frontal area is related to driver distraction and represents the strength of distraction in real-life situations.

## Background

Driver distraction has been identified as the leading cause of car accidents. The U.S. National Highway Traffic Safety Administration had reported driver distraction as a high priority area about 20-30% of car accidents [1]. Distraction during driving by any cause is a significant contributor to road traffic accidents [2,3]. Driving is a complex task in which several skills and abilities are simultaneously involved. Distractions found during driving are quite widespread, including eating, drinking, talking with passengers, using cell phones, reading, feeling fatigue, solving problems, and using in-car equipment. Commercial vehicle operators with complex in-car technologies also cause an increased risk as they may become increasingly distracting in the years to come [4,5]. Some literature studied the behavioral effect of driver's distraction in car. Tijerina showed driver distraction from measurements of the static completion time of an in-vehicle task [6]. Similarly, distraction effects caused by talking on cellular phones during driving have been a focal point of recent in-car studies [7-9]. Experimental studies have been conducted to assess the impact of specific types of driver distraction on driving performance. Though these studies generally reported significant driving impairment, simulator studies cannot provide information about accidents due to impairment resulting in hospitalization of the driver [10,11]. To provide information before the occurrence of crashes, the drivers' physiological responses are investigated in this paper. However, monitoring drivers' attention-related brain resources is still a challenge for researchers and practitioners in the field of cognitive brain research and human-machine interaction.

Regarding neural physiological investigation, some literature focused on the brain activities of "divided attention," referring to attention divided between two or more sources of information, such as visual, auditory, shape, and color stimuli. Positron emission tomography (PET) measurements were taken while subjects discriminated among shape, color, and speed of a visual stimulus under conditions of selective and divided attention. The divided attention condition activated the anterior cingulated and prefrontal cortex in the right hemisphere [12]. In another study, functional magnetic resonance imaging (fMRI) was used to investigate brain activity during a dual-task (visual stimulus) experiment. Findings revealed activation in the posterior dorsolateral prefrontal cortex (middle frontal gyrus) and lateral parietal cortex [13]. In addition, several neuroimaging studies showed the importance of the prefrontal network in dual-task management [14,15]. Some studies investigated traffic scenarios recorded the EEG to compare P300 amplitudes [16]. During simulated traffic scenarios, resource allocation was assessed as an event-related potential (ERP) novelty oddball paradigm [17]. In these EEG studies, however, only the time course was analyzed. Deiber took one more step to analyze the relation between time and frequency courses [18]. Their study used EEG to investigate mental arithmetic-induced workload and found theta band power increases in areas of the frontal cortex. Despite so much research on brain activities, the above-mentioned studies only investigated brain activities during dual-task interactions without considering the SOA problem during driving, which is with the temporal gap between presentations of two stimuli. When dual tasks are presented within a short SOA, the response time of each task is typically lower than that presented within a longer SOA [19]. Therefore, the current study investigates the effects of the different temporal relationships of stimuli.

Clinical practices as well as basic scientific studies have been using the EEG for 80 years. Presently, EEG measurement is widely used as a standard procedure in research such as sleep studies, epileptic abnormalities, and other disorder diagnoses [20,21]. Compared to another widely used neuroimaging modality, fMRI, the EEG is much less expensive and has superior temporal resolution in investigating SOA problems. To avoid interference and decrease risks while operating a vehicle on the road, researchers adopted driving simulations for vehicle design. Studies of driver's behavior and cognitive states are also expanding rapidly [22]. However, static driving simulation cannot fully create real-life driving conditions, such as the vibrations experienced when driving an actual vehicle on the road. Therefore, the VR-based simulation with a motion platform was developed [23,24]. This VR technique allows subjects to interact directly with a virtual environment rather than only monotonic auditory or visual stimuli. Integrating realistic VR scenes with visual stimuli makes it easy to study the brain response to attention during driving. Therefore, in recent years, VR-based simulation combined with EEG monitoring is a recent and beneficial innovation in cognitive engineering research.

The main goal of this study is to investigate the brain dynamics related to distraction by using EEG and a VR-based realistic driving environment. Unlike previous studies, the experiment design has three main characteristics. First, the SOA experimental design, with different appearance times of two tasks, has the benefit of investigating the driver's behavioral and physiological response under multiple conditions and multiple distraction levels. Second, ICA-based advanced analysis methods are used to extract brain responses and the cortical location related to distraction. Third, this study investigates the interaction and effects of dual-task-related brain activities, in contrast to a single task.

## Methods

### Subjects

Fifteen healthy participants (all males), between 20 and 28 years of age, were recruited from the university population. They have normal or corrected-to-normal vision, are right handed, have a driver's license, and are reported being free from psychiatric or neurological disorders. Written informed consent was obtained prior to the study.

Each subject participated in four simulated sessions inside a car with hands on the steering wheel to keep the car in the center of the third lane, which was numbered from the left lane, in a VR surround scene on a four-lane freeway [23]. Thirty scalp electrodes (Ag/AgCl electrodes with a unipolar reference at the right earlobe) by the NuAmp system (Compumedics Ltd., VIC, Australia) were mounted on the subject's head to record the physiological EEG [25]. The EEG electrodes were placed based on a modified international 10-20 system. The contact impedance between EEG electrodes and the cortex was calibrated to be less than 10 kΩ. Before beginning first session, each subject took a 15 ~ 30 minute for practice session. In each session, subjects proceeded to a freeway simulated driving lasting fifteen minutes with the corresponding EEG signals synchronously recorded. For these four-session experiments, subjects were required to rest for ten minutes between every two sessions to avoid fatigue.

### Recordings and experimental conditions

For this study, a simulated freeway scene was built using VR technology with a WTK library on a 6 DOF motion platform [23]. The four-lane freeway scene was displayed on a surrounded environment. Since the main purpose of this paper is to investigate distraction effects in dual-task conditions, two tasks involving unexpected car deviations and mathematical questions were designed. In the driving task, the car frequently and randomly drifted from the center of the third lane. Subjects were required to steer the car back to the center of the third lane. This task mimicked the effects of driving on a non-ideal road surface. In the mathematical task, two-digit addition equations were presented to the subjects. The answers were designed to be either valid or invalid. Subjects were asked to press the right or left button on the steering wheel corresponding to on correct or incorrect equations, respectively. The allotment ratio of correct-incorrect equations was 50-50. The choice of mathematic task was motivated by the desire for control in the task demands [26]. All drivers could perform this mathematic task well without training.

**Figure 1:**
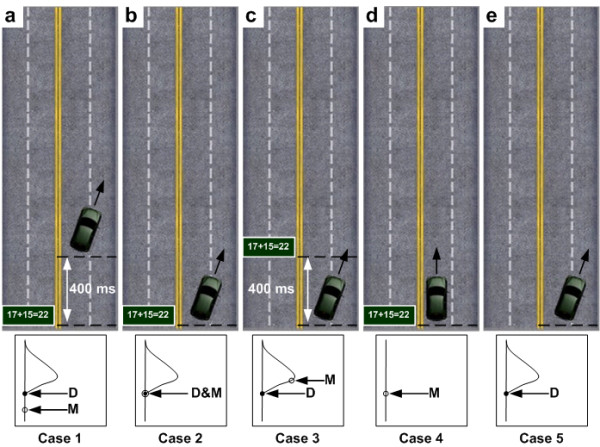
**The illustration shows the relationship of occurrences between the deviation and math tasks**. D: deviation task onset. M: math task onset. (a) Case 1: math task presents 400 ms before the deviation task onset. (b) Case 2: math and deviation tasks occur at the same time. (c) Case 3: math task presents 400 ms after the deviation task onset. (d) Case 4: only math task presents. (e) Case 5: only deviation task occurs. The bottom insets show the onset sequences of the two tasks.

To investigate the effects of SOA between two tasks, the combinations of these two tasks were designed to provide different distracting conditions to the subjects as shown in Figure 1. Five cases were developed to study the interaction of the two tasks. The bottom insets show the onset sequences of two tasks. Therefore, this study investigated the relationship of math task and driving task and how two tasks affected each other in the SOA conditions.

### Statistical analysis of behavior performance

After recording the behavior data, statistical package for the social science (SPSS) Version 13.0 for Windows software is applied to estimate the significance testing of behavior data. The response time of these two tasks (the driving deviation and the math equation) is analyzed to study the behavior of subjects in the experiments.

Using ANOVA (analysis of variance), the significances of the response time of these two tasks are tested for every subject. A non-parametric test is also utilized to study the trends of the behavior data. Firstly, this study excluded outliers, comprising around 6.57% of all trials, based on the criteria that response time was distributed outside the mean response time plus three times the standard deviation of each single session. Secondly, the number of trials in one of five cases which is minimal is chosen to make a benchmark to randomly select the same number of trials in other cases. Thirdly, a single task is taken for the baseline to normalize the behavior data to be $\frac{\text{Xi}}{\text{Xmean}}$ (Xi: mean of response time in case i, Xmean: mean of response time in single case). For example, in order to compare the distraction effects from the math equation, case 4 (the single math task) is the baseline.

### Measurement of distraction effects in dual-task EEG time series

EEG epochs are extracted from the recorded EEG signals with 16-bit quantization, at the sampling rate of 500 Hz. The data are then preprocessed using a simple low pass filter with a cut-off frequency of 50 Hz to remove line noise and other high frequency noise. One more high-pass filter with a cut-off frequency of 0.5 Hz is utilized to remove DC drift. This study adopts ICA to separate independent brain sources [27-29]. ERSP technology is then applied to these independent component (IC) signals (separated independent brain sources) to transfer the signal into the time-frequency domain for the event-related frequency study. Finally, the stability of component activations and scalp topographies of meaningful components are investigated with component clustering technology. Because different cases with various combinations of driving and the math tasks are designed, EEG responses from five different cases are extracted separately.

EEG source segregation, identification, and localization is very difficult because EEG data collected from the human scalp induce brain activities within a large brain area. Although the conductivity between the skull and brain is different, the spatial "smearing" of EEG data caused by volume conduction does not cause a significant time delay. This suggests that ICA algorithm is suitable for performing blind source separation on EEG data. The first applications of ICA to biomedical time series analysis were presented by Makeig and Inlow [30]. Their report shows segregation of eye movements from brain EEG phenomena, and separates EEG data into constituent components defined by spatial stability and temporal independence. Subsequent technical experiments demonstrated that ICA could also be used to remove artifacts from both continuous and event-related (single-trial) EEG data [27,28]. Presumably, multi-channel EEG recordings are mixtures of underlying brain sources and artificial signals. By assuming that (a) mixing medium is linear and propagation delays are negligible, (b) the time courses of the sources are independent, and (c) the number of sources is the same as the number of sensors; that is, if there are N sensors, the ICA algorithm can separate N sources [27].

The time sequences of ICA component signals are subjected to Fast Fourier Transform with overlapped moving windows. In addition, the spectrum in each epoch is smoothed by 3-window (768 points) moving-average to reduce random errors. The spectrum prior to event onsets is considered as the baseline spectrum for every epoch. The mean of the baseline spectrum is subtracted from the power spectral after stimulus onsets so spectral "perturbation" can be visualized. This procedure is then applied repeatedly to every epoch. The results are averaged to yield ERSP images [31]. These measures can evaluate averaged dynamic changes in amplitudes of the broad band EEG spectrum as a function of time following cognitive events. The ERSP images mainly show spectral differences after an event since the baseline spectrum prior to event onsets had been removed. After performing a bootstrap analysis (usually 0.01 or 0.03 or 0.05; here 0.01 was applied) on ERSP, only statistically significant (p < 0.01) spectral changes are shown in the ERSP images. Non-significant time/frequency points are masked (replaced with zero). Consequently, any perturbations in the frequency domain become relatively prominent.

To study the cross-subject component stability of ICA decomposition, components from multiple subjects are clustered, based on their spatial distributions and EEG characteristics. However, components from different subjects differ in many ways such as scalp maps, power spectrum, ERPs and ERSPs. Some studies attempted to solve this problem by calculating similarities among different ICs [32-34]. Based on these studies, ICs of interest are selected and clustered semi-automatically based on their scalp maps, dipole source locations, and within-subject consistency. To match scalp maps of ICs within and across subjects in this paper, the gradients of the IC scalp maps from different sessions of the same subject are computed and grouped together based on the highest correlations of gradients of the common electrodes retained in all sessions. For dipole source locations, DIPFIT2 routines from EEGLAB are used to fit single dipole source models to the remaining IC scalp topographies using a four-shell spherical head model [35]. In the DIPFIT software, the spherical head model is co-registered with an average brain model (Montreal Neurological Institute) and returns approximate Talairach coordinates for each equivalent dipole source.

## Results

### Behavior performance

**Figure 2:**
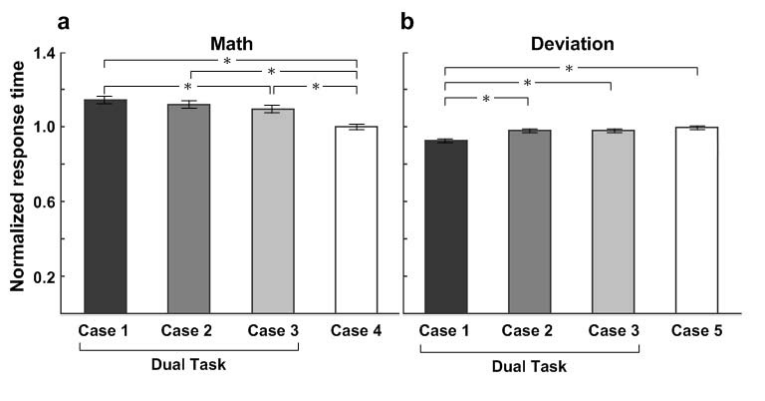
**This shows the bar charts of normalized response times**. (a) for the math task and (b) for deviation task across 15 subjects. The filled black bar: case 1; dark gray bar: case 2; light gray bar: case 3; the open bar: single case. The response time for math task in dual-task cases (case 1, case 2, and case 3) is significantly longer than that for in single task (case 4). The shortest response time for the math onset is in case 4. The response time for deviation task in case 1 is significantly shorter than those in other cases. The longest response time to the deviation onset is in case 5. The bottom insets show the onset sequences of the two tasks.

To investigate the overall behavior index, this study uses nonparametric tests because several extremely large scores are significantly skewed. Firstly, the trials of data are randomly selected to have the same number of the trials in all cases. Then, the response time of the deviation and math tasks in the five cases are normalized to correspond to single-deviation and single-math cases, respectively. SPSS software is used for the Friedman test, and the results of which are shown in Figure 2. Dual-task cases are marked for easy discrimination from single-task cases.

To know how the cases make the differences, the Student-Newman-Keuls test is used for the post hoc test (in Table 1). The test statistic on response time of math tasks in cases 1-4, is χ^2^(3) = 903.926 from the Friedman's ANOVA test, and p < 0.01. The Student-Newman-Keuls test show three significant groups: case 1 with case 2, case 3, and case 4 in which the response time for math task in case 1 is the longest. Statistical test results of the response time for deviation tasks in cases 1-3, and case 5, is χ^2^(3) = 493.98 from the Friedman's ANOVA test, and p < 0.01. Using the Student-Newman-Keuls test, there are two significant groups: case 1, and the other cases in which the response time for deviation task in case 1 is the shortest.

**Table 1:** The normalized response time to deviation and math

Case	Response time to deviation			Response time to math		
		
	Mean	Standard deviation	Difference (dual-single)	Mean	Standard deviation	Difference (dual-single)
Case 1	0.9480	0.1314	*p < 0.01*	1.1479	0.3061	*p < 0.01*
Case 2	0.9856	0.1269	*p > 0.01*	1.1277	0.2724	*p < 0.01*
Case 3	0.9865	0.1231	*p > 0.01*	1.0975	0.2727	*p < 0.01*
Single (baseline)	1	0.1553		1	0.2168	

### Independent component clustering

EEG epochs are extracted from the recorded EEG signals. Then, ICA is utilized to decompose independent brain sources from the EEG epochs. Based on distraction effects in this study, many brain resources are involved in this experiment. Especially, the motor component is active when subjects are steering the car. At the same time, activations related to attention in the frontal component appear. Therefore, ICA components, including frontal and motor, are selected for IC clustering to analyze cross-subject data based on their EEG characteristics.

**Figure 3:**
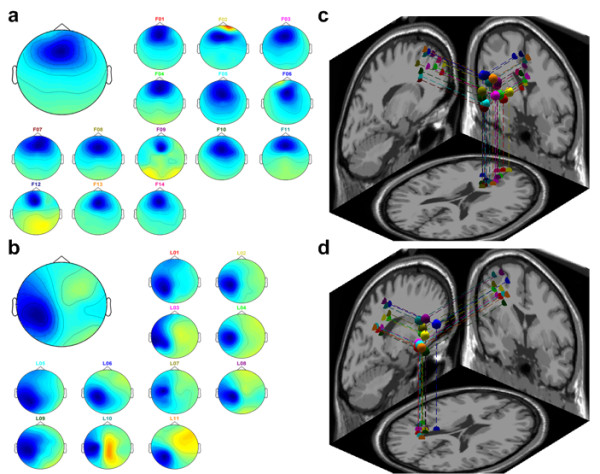
**The scalp maps and equivalent dipole source locations after IC clustering across 15 subjects**. (a) the frontal components and (b) the left motor components are shown here. There are 14 subjects in the frontal cluster and 11 subjects in the left motor cluster. The grand scalp map is the mean of the total component maps in each cluster. The smaller maps are the individual scalp maps. The right panels (c) and (d) show the 3-D dipole source locations (colored spheres) and their projections onto average brain images. The colored source locations correspond to their own scalp maps by the same color of the text above.

At first, IC clustering groups massive components from multiple sessions and subjects into several significant clusters. Cluster analysis, k-means, is applied to the normalized scalp topographies and power spectra of all 450 (30 channels × 15 subjects) components from the 15 subjects. Cluster analysis identifies at least 7 component clusters having similar power spectra and scalp projections. These 7 distinct component clusters consisted of frontal, central midline, parietal, left/right motor and left/right occipital. Table 2 gives the number of components in different clusters. This investigation uses the frontal and left motor components to analyze distraction effects. Figure 3 shows the scalp maps and equivalent dipole source locations for fontal and left motor clusters. Based on this finding, the EEG sources of different subjects in the same cluster are from the same physiological component.

**Table 2:** The Number of Components in Different Clusters

	Frontal	Central Midline	Parietal	Left Motor	Right Motor	Left Occipital	Right Occipital
Number of components	14	12	9	11	8	6	4

### Frontal and left motor clusters

**Figure 4:**
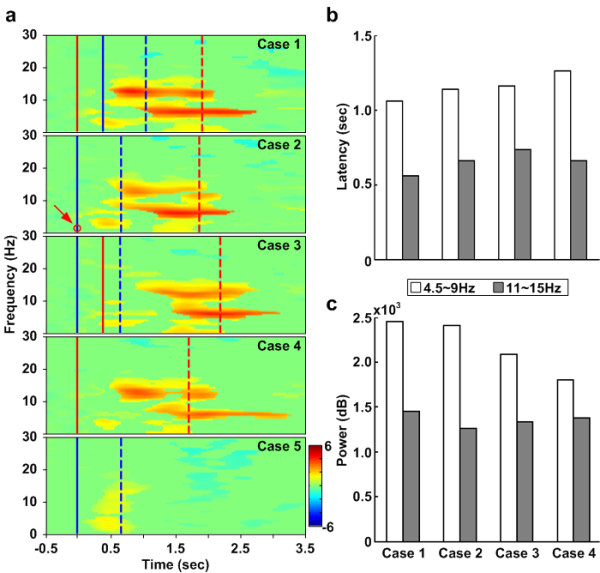
**The ERSP images of frontal cluster with five cases**. (a) The ERSP images of frontal cluster with five cases. The right column show the onset sequences of the two tasks. Color bars indicate the magnitude of ERSPs. Red solid lines show the onset of the math task. Red dashed lines show the mean response time for the math task. Blue solid lines show the onset of the deviation task. Blue dashed lines show the mean response time for the deviation task. The red circle pointed out by the red arrow in case 2 means the red solid line and blue solid line are on the same position. Latencies calculated from (a) are shown in (b) by calculating time form the math task onset to the first occurrence of power increases. The open bars represent the latencies in the theta (4.5 ~ 9 Hz) band. The gray bars represent these latencies in the beta (11 ~ 15 Hz) band. The comparison of total power in cross-subject (14 subjects) averaged ERSP images in the frontal cluster between cases is shown in (c). The amount of total power is calculated by adding all the power increases in the same temporal period and the same frequency band. The open bars represent the total power in the theta band. The gray bars represent the total power in the beta band.

Figure 4a shows the cross-subject averaged ERSP in the frontal cluster corresponding to the five cases. Figure 4 also reveals significant (p < 0.01) power increases related to the math task, demonstrating that the power increases in the frontal cluster are related to the math task. The theta power increases in three dual-task cases including cases 1-3 are slightly different from each other. Compared to the single math task (case 4), the power in dual-task cases is stronger. Especially, the power increase in case 1 is the strongest. On the beta band, it also shows power increases, which appear only in the math-task and time-locked to mathematics onsets.

Figure 4b and 4c give comparisons of the latency and total power in four cases from Figure 4a. It demonstrates that the latencies of power increases in two frequency bands are different with the different SOA time. The shortest latencies in both bands occur in case 1 and the longest power increase latency in the theta band occurs in case 4. It also demonstrates that the amount of power increases in the theta band is different with the different SOA time. The most significant power increase occurs in case 1.

**Figure 5:**
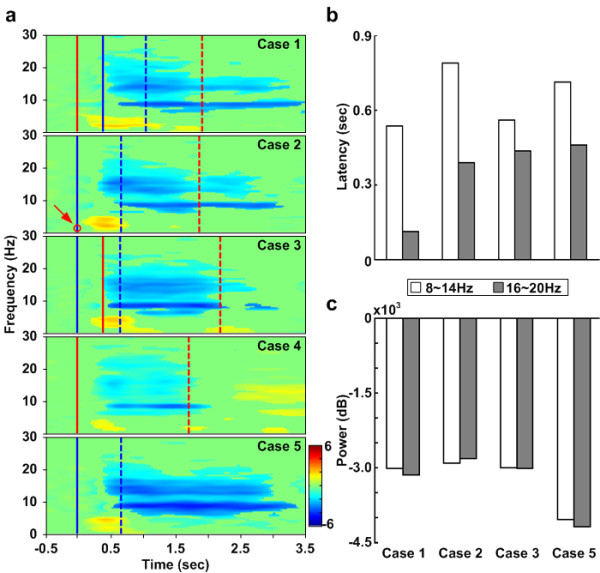
**The ERSP images of the left motor cluster with five cases**. (a) The ERSP images of the left motor cluster with five cases. The right column shows the onset sequences of the two tasks. Color bars indicate the magnitude of the ERSPs. Red solid lines show the onset of math. Red dashed lines show the mean response time for math task. Blue solid lines show the onset of deviation task. Blue dashed lines show the mean response time for deviation task. The red circle pointed out by a red arrow in case 2 means the red solid line and blue solid line are on the same position. Latencies calculated from (a) are shown in (b) by calculating from the deviation task onset to the first occurrence of power suppressions. The open bars represent the latencies in the alpha (8 ~ 14 Hz) band. The gray blue bars represent these latencies in the beta band (16 ~ 20 Hz). (c) shows the comparison of total power in cross-subject (11 subjects) averaged ERSP images in the left motor cluster between cases. The amount of total power is calculated by adding all the power suppressions in the same temporal period and the same frequency band. The open bars represent the total power in the alpha band. The gray bars represent the total power in the beta band.

Figure 5a shows the cross-subject average ERSP in the left motor cluster corresponding to five cases. Significant (p < 0.01) power suppressions appear around the event onsets (at 0 ms) and stop at different time axes by cases. In case 4, the alpha and beta power suppressions appear continuously until the red dashed lines, which indicates the mean of the response time for the math task. Compared with case 4, the alpha and beta power suppressions in case 5 are stronger and also last longer. In other cases, the alpha and beta power suppressions continue after the blue dashed lines. This phenomenon is suggested to be related to steering the car back to the center of the third lane.

Figure 5b and 5c shows comparisons of the latency and total power between the four cases in Figure 5a. It demonstrates that power suppression latencies in the beta band are different with the different SOA time. The shortest power suppression latency occurs in case 1 and the longest power increase latency occurs in case 5. It also demonstrates that the amount of power suppression in the alpha band is different with the different SOA time. The most significant power suppression occurs in case 5 (the single driving task) and the smallest power suppression occurs in case 4 (the single math task).

**Figure 6:**
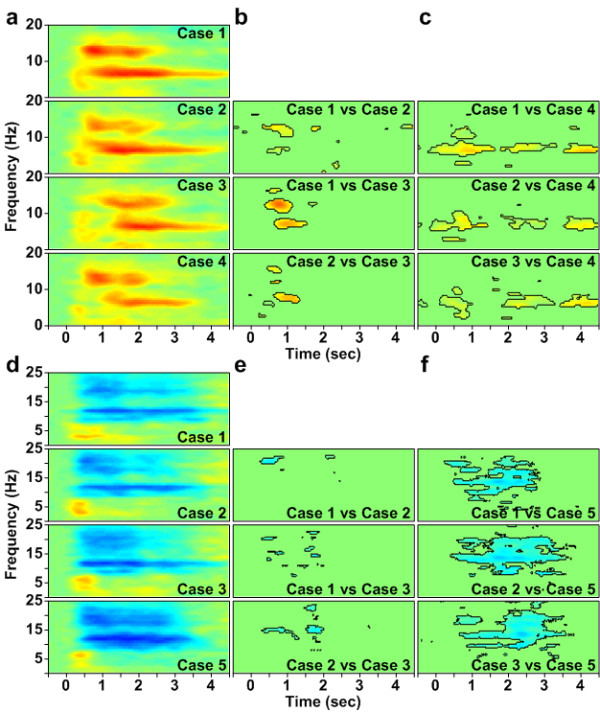
**ERSP without a significance test and the differences between cases**. Column (a) shows the ERSP in the frontal cluster without a significance test which contains all the details of case 1, case 2, case 3, and case 4. Column (b) shows the differences among three single-task cases in column (a). Column (c) shows the differences between single- and dual-task cases in column (a). Column (d) shows the ERSP in the left motor cluster without a significance test which contains all the details of case 1, case 2, case 3, and case 5. Column (e) shows the differences among three single-task cases in column (d). Column (f) shows the differences between single- and dual-task cases in column (d). A Wilcoxon signed-rank test (p < 0.01) is used for the statistical test in (b), (c), (e), and (f).

Figure 6a and 6d show the ERSP in the frontal and left motor clusters without a significance test. Columns (b) and (e) show the differences among three single-task cases; columns (c) and (f) show the differences between single- and dual-task cases. In columns (b), (c), (e), and (f), a Wilcoxon signed-rank test is used to retain the regions with significant power inside the black circles. Columns (b) and (c) show the comparison of power increases between cases. The remained regions show greater power increases in the single-task case than in the dual-task case. Columns (e) and (f) show compared power suppressions between cases. The remained regions show greater power suppressions in the dual-task cases than in the single-task case.

## Discussion

### Frontal cluster

The frontal lobe is an area in the brain, located at the front of each cerebral hemisphere. The frontal area deals with impulse control, judgment, language production, working memory, motor function, and problem solving [36,37]. In Figure 4a, the greater frontal power increases in cases 1-4 appear due to the solving of the math questions. The power increases in the theta (4.5 ~ 9 Hz) and beta bands (11 ~ 15 Hz) appear briefly after the math onset. Figure 4b and 4c show the quantified frontal power latencies and power increases in four conditions for the purpose of discussing the EEG dynamics made by solving the math question. In the theta power, the shortest latency is revealed in case 1. Power increases in three dual-task cases are higher than that in single-task case with the greatest power occurring in case 1. These phenomena suggest that dual tasks induce more event-related theta activities as well as subjects need more brain resources to accomplish dual tasks. The theta increase is associated with numerous processes such as mental work load, problem solving, encoding, or self monitoring [34]. Based on this evidence, the study demonstrates that the subjects were distracted under dual-task conditions in the experiment.

Since human visual sensors need about 300 ms to perceive stimulus (P300 activity), 400 ms between first and second tasks is sufficient for a subject to perceive stimulus[38]. In case 1, a processing task is already in the brain and subjects need more brain resources to manage the high priority task presented 400 ms after the processing task. Therefore, the total power in the theta band in case 1 is the highest as shown in Figure 4c. Clearly the theta power increase appears the earliest in case 1 as shown in Figure 4b. The early theta response in the frontal area primarily reflects the activation of neural networks involved in allocating attention related to the target stimulus [39].

The trends of response time for the math task (in Figure 2a) and EEG theta increases in the frontal cluster (in Figure 4c) are consistent with one another. In the case of the single math task, the response time is the shortest and the theta power increase is the weakest. Among the dual-task cases, the longest response time and the greatest theta power increase are in case 1. This evidence suggests that the theta activity of the EEG in the frontal area during dual tasks is related to distraction effects and represents the strength of distraction. In addition, power increases in the beta band appear in all cases. From the ERSP images, the patterns are time-locked to the onset of the math task. Fernández suggested that significant EEG beta band differences in the frontal area are due to a specific component of mental calculation [40].

### Motor cluster

Mu rhythm (μ rhythm) is an EEG rhythm usually recorded from the motor cortex of the dominant hemisphere. It can be suppressed by simple motor activities such as clenching the fist of the contra lateral side, or passively moved [41-43]. Mu suppression is believed to be the electrical output of the synchronization on large portions of pyramidal neurons in the motor cortex that controls hand and arm movements.

In this study, the mu suppressions (8 ~ 14 Hz) and beta power suppression (16 ~ 20 Hz) are mostly caused by subjects steering the wheel and pressing buttons as shown in Figure 5a. The mu suppressions caused by steering the wheel are almost time-locked to the response onset of driving task in cases 1-3 and case 5. However, the mu suppressions caused by pressing the buttons have no effects in case 4. As for in the dual-task cases, the mu suppressions are weaker than those in single-task case. This may due to the competition of brain resources required by wheel steering and button pressing.

Thus, Figure 5b and Figure 5c show motor power latencies and power increases in 4 cases for the purposes of discussing the EEG dynamics caused by the driving task. In (b), the longest latency of beta power suppression is observed in case 5 and the shortest latency appears in case 1. Perhaps motor planning is involved in preparing for steering the wheel and answering the math questions [44]. In (c), the three dual-task power suppressions are weaker than those in single task. Based on above evidences, it suggests that math processing occupies more brain resources in the frontal area during dual-task cases so less activation is induced in the motor area.

### Brain dynamics related to behavior performance

Posner postulated that two tasks performed simultaneously did not interfere with each other's performance when different brain areas were used for these two tasks [45]. However, this study uses two visual-stimuli tasks that compete within the frontal and motor areas for taking action. From the results, these two visual-stimuli tasks interfere with each other in both behavior performance (in Figure 2) and brain dynamics (in Figure 6).

In order to compare brain dynamics among different cases (in Figure 6), a statistical analysis was also conducted to assess the significance of the ERSP differences of the independent clusters under different cases. Since the true sample distribution of the cluster ERSP was unknown and the sample size (N = 14 as 1 of 15 subjects and N = 11 as 4 of 15 subjects were exclude in frontal and left motor clusters, respectively) was small, a nonparametric statistical analysis, a paired-sample Wilcoxon signed-rank test, was employed to access the statistically significant ERSP differences under different cases. The level of significance was set to p < 0.01.

In Figure 6c, the significant differences between dual-task cases and case 4 are due to that subjects' reaction to a math question is impaired when they are also facing a car deviation. Lavie demonstrated that dual-task load increases distraction effects [46]. Because of the distraction effects, the behavioral response time are significantly higher in dual-task cases than that in single-task case. In order to study the comparisons of these dual-task cases, the differences of them are shown in Figure 6b. From the behavior performance in Figure 2, response time in case 1 and case 2 are the longest which means that the most distraction effects occurred in these two cases. It is also shown in Figure 6b. Especially, distraction effects in case 1 are slightly higher than those in case 2. Therefore, it is suggested that some kinds of two sequent tasks make the same distraction effects as two simultaneous tasks, or even higher.

Jong investigated how performance of two overlapping discrete tasks was organized and controlled [47]. The sequential performance of overlapping tasks can be scheduled in advance and regulated by initially allocating brain resources to one task and subsequently switching to the other task. Thus in case 1, when the math task is presented to the subject, it occupies the brain resources. Then because the driving task appears, the brain resources are immediately switched to the driving task and the math task is temporally dropped. Subsequently, the brain resources are then switched back to the math task. This processing consumes the most brain resources and makes the longest response time for the math question The response time in case 1 is significantly higher than that in case 3 and case 4. The occurrence of distraction effects is due in large part to the switching of brain resources.

The fact, which no significant differences occur on behavior performance for the driving tasks between the simultaneous-task case 2 and single-task case 5 (in Figure 2), suggests that the driving task is too simple to require much brain resources. These results are also due to the first priority on the driving task. No differences of behavior performance, which appear among case 2, case 3 and case5, also prove this fact. Thus, the subjects always chose to respond to the driving task when the driving task occurs even if they are handling a math task. In case 1, however, the math question is took as a cue to let the subjects rapidly respond to the driving task to avoid hitting the wall. This situation makes the response time short for the driving task in case 1 due to the subjects under a high perceptual load. Consistently, Lavie demonstrated that a high perceptual load reduced response time [46]. This also causes case 1 and case 3, which are formed as a symmetrical paradigm, be much different from each other (in Figure 2).

In Figure 6, the most power suppression occurs in case 5 (in Figure 6f) with only driving task. Three dual-task cases have the same level of power suppression. The reason why less power suppression occurs on dual-task cases in motor area is suggested that most brain resources are occupied in frontal area to deal with two tasks instead of those in motor area. It is proposed that motor area is not related to distraction effects. This is proved by one more result that the correlation is low between EEG dynamics in motor area and its corresponding response time.

In summary, this study observes several differences between dual-task and single-task cases. We investigate the relationship between brain dynamics associated with dual-task management and the behavior performance of response modalities. It is suggested the theta activity of the EEG in the frontal area during dual tasks is related to distraction effects and represents the strength of distraction. In addition, the appearing order of the two tasks with different difficulties is an important factor in dual-task performance.

## Conclusions

This study investigates behavioral and physiological (EEG) responses under multiple cases and multiple distraction levels. Firstly, the response time for mathematical problem solving in dual-task condition is significantly higher than that in single-task condition. Therefore, distraction effects occur while processing two tasks during driving. Comparing to the mathematical problems, however, the response time for driving tasks under multiple cases is almost the same without differences. This is due to the order of task appearance and the relative difficulty of the two tasks, which suggesting these factors are important considerations in dual-task performance. Secondly, theta power increases in the frontal area are higher with higher response time. The phasic changes around the theta band in the case, in which the mathematic task is presented before the deviation task, show the strongest increase as the same as that in the simultaneous-task case. This is because subjects already process a task in the brain and need more brain resources to manage the second task presented after the first task. In conclusions, this study suggests that the power increases of the 4.5 ~ 9 Hz frequency band in the frontal area is related to driver distraction and represents the strength of distraction in real-life driving.

## Competing interests

The authors declare that they have no competing interests.

## Authors' contributions

CTL started this study with the main idea, participated in the design of this study, and led the team to well finish it. SAC participated in the design of the study, the acquisition of data, the analysis/interpretation of data, and the modification of paper to submit. TTC participated in the design of the study and performed the statistical analysis. HZL participated in the design of the study and drafted the manuscript. LWK conceived of the study, and participated in the design and coordination and helped to draft the manuscript. All authors read and approved the final manuscript.
